# Consensus on the diagnosis and management of chronic leg ulcers - Brazilian Society of Dermatology^☆^^☆☆^

**DOI:** 10.1016/j.abd.2020.06.002

**Published:** 2020-10-04

**Authors:** Luciana Patricia Fernandes Abbade, Marco Andrey Cipriani Frade, José Roberto Pereira Pegas, Paula Dadalti-Granja, Lucas Campos Garcia, Roberto Bueno Filho, Carlos Eduardo Fonseca Parenti

**Affiliations:** a Department of Infectious Diseases, Dermatology, Diagnostic Imaging and Radiotherapy, Faculty of Medicine, Universidade Estadual Paulista, Botucatu, SP, Brazil; b Department of Internal Medicine (Dermatology Division), Faculty of Medicine, Universidade de São Paulo, Ribeirão Preto, SP, Brazil; c Dermatology Service, Hospital Padre Bento de Guarulhos, Guarulhos, SP, Brazil; d Discipline of Dermatology, Faculty of Medicine, Universidade da Cidade de São Paulo, São Paulo, SP, Brazil; e Discipline of Dermatology, Faculty of Medicine, Jundiaí, SP, Brazil; f Department of Clinical Medicine (Discipline of Dermatology), Universidade Federal Fluminense, Niterói, RJ, Brazil; g Dermatology Service, Hospital das Clínicas, Universidade Federal de Minas Gerais, Belo Horizonte, MG, Brazil; h Dermatology Service, Hospital das Clínicas, Faculty of Medicine, Universidade de São Paulo, Ribeirão Preto, SP, Brazil; i Department of Dermatology, Universidade Federal de São Paulo, São Paulo, SP, Brazil

**Keywords:** Cutaneous ulcer, Diabetes mellitus, Leg ulcer, Leprosy, Neuropathy, Peripheral arterial disease, Venous ulcer

## Abstract

**Background:**

Chronic leg ulcers affect a large portion of the adult population and cause a significant social and economic impact, related to outpatient and hospital care, absence from work, social security expenses, and reduced quality of life. The correct diagnosis and therapeutic approach are essential for a favorable evolution.

**Objective:**

To gather the experience of Brazilian dermatologists, reviewing the specialized literature to prepare recommendations for the diagnosis and treatment of the main types of chronic leg ulcers.

**Methods:**

Seven specialists from six university centers with experience in chronic leg ulcers were appointed by the Brazilian Society of Dermatology to reach a consensus on the diagnosis and therapeutic management of these ulcers. Based on the adapted DELPHI methodology, relevant elements were considered in the diagnosis and treatment of chronic leg ulcers of the most common causes; then, the recent literature was analyzed using the best scientific evidence.

**Results:**

The following themes were defined as relevant for this consensus - the most prevalent differential etiological diagnoses of chronic leg ulcers (venous, arterial, neuropathic, and hypertensive ulcers), as well as the management of each one. It also included the topic of general principles for local management, common to chronic ulcers, regardless of the etiology.

**Conclusion:**

This consensus addressed the main etiologies of chronic leg ulcers and their management based on scientific evidence to assist dermatologists and other health professionals and benefit the greatest number of patients with this condition.

## Introduction

The prevalence and incidence of chronic ulcers is increasing with the aging of the population and higher prevalence of associated chronic conditions, such as systemic arterial hypertension and diabetes mellitus.^1^ Many diseases manifest themselves as chronic ulcers, especially those of the legs, which occur below the knee, do not heal within six weeks, and cause a significant social and economic impact.^2^

The most common etiologies are venous, arterial, and neuropathic, corresponding to 90% of the causes; however, the hypertensive etiology is also relatively frequently. These ulcers will be discussed in this consensus, focusing on the diagnosis and specific management of each etiology, and the general principles for their approach, with the recommendations of Brazilian specialists in dermatology.

## Methods

Seven specialists from six university centers with experience in chronic leg ulcers were appointed by the Brazilian Society of Dermatology to reach a consensus on the diagnosis and therapeutic management of these ulcers using the adapted DELPHI methodology. In the first phase, the topics that would be addressed were defined; subsequently, the themes were divided by expertise for a search in the recent literature, with emphasis on treatment recommendations available in Brazil. Consensus was defined as approval by at least 70% of the panel members.

## Results and discussion

The following themes were defined as relevant for this consensus: the most prevalent differential etiological diagnoses of chronic leg ulcers, including venous, arterial, neuropathic, and hypertensive ulcers. It was also decided to address the topic of general principles for the local management of injuries, as they are common to all chronic ulcers, regardless of their etiology.

Table 1 lists the causes of chronic skin ulcers, with emphasis on those that occur on the legs.

**Table 1:** Main causes of chronic skin ulcers.

Infectious	Bacterial (bullous erysipelas, necrotizing fasciitis, botryomycosis, gas gangrene, ecthyma gangrenosum, septic embolism, bacterial endocarditis, carbuncle [*Bacillus anthracis*], diphtheria, meningococcemia, bartonellosis, glanders, tularemia, and yaws)
	Mycobacteriosis (leprosy, Buruli ulcer, tuberculosis)
	Viral (herpes simplex, varicella zoster, cytomegalovirus)
	Fungal (bullous tinea pedis, eumycotic mycetoma, chromomycosis, coccidioidomycosis, sporotrichosis, histoplasmosis and paracoccidioidomycosis)
	Protozoa (leishmaniasis, amoebiasis)
Drug-induced	Hydroxyurea, methotrexate, chemotherapy, warfarin
Neoplastic	Metastasis of internal malignancies, squamous cell carcinoma (Marjolin's ulcer)
	Basal cell carcinoma;
	Melanoma, Merkel carcinoma, Kaposi's sarcoma
	Malignant fibrous histiocytoma, lymphoproliferative diseases
Systemic diseases	aDiabetes mellitus
	Neuropathic (tabes dorsalis, paraplegia, multiple sclerosis)
	Genetic (Klinefelter syndrome)
	aArterial hypertension (Martorell's ulcer)
	Hematological (polycythemia vera, sickle cell anemia, thrombocytopenia, paraproteinemia)
	Autoimmune (scleroderma, rheumatoid arthritis, lupus erythematosus)
	Inflammatory (inflammatory bowel disease, including metastatic Crohn's disease)
	Nutritional deficiencies (caloric, protein, vitamins, and minerals)
Primary skin diseases	Necrobiosis lipoidica, sarcoidosis
	Pyoderma gangrenosum
	Panniculitis (including erythema induratum)
	Bullous (pemphigus, bullous pemphigoid, bullous lichen planus, porphyria cutanea tarda) Stevens-Johnson syndrome, and toxic epidermal necrolysis
Related to drug abuse	Injection of illicit drugs, toxic and irritating effects of illicit or adulterated drugs, cocaine-induced vasoconstriction, bacterial embolism
Trauma	Burns, bites, post-surgical
Factitious	Dermatitis artefacta, disease simulation, Munchausen syndrome
Vascular	aVenous ulcers: chronic venous insufficiency, congenital valve insufficiency, post-traumatic valve insufficiency, mixed venous-arterial or venous-lymphatic insufficiency, arteriovenous malformation
	a Arterial ulcers and thromboangiitis obliterans
Vasculitis	Small vessel vasculitis: leukocytoclastic, microscopic polyangiitis, granulomatosis with polyangiitis (Wegener's granulomatosis), Churg-Strauss, Henoch-Schönlein purpura, cryoagglutination (cryoglobulinemia, cryofibrinogen), and Behçet's disease
	Medium-size-vessel vasculitis: polyarteritis nodosa, anti-phospholipid antibody syndrome
Vasculopathy	Hypercoagulability disorders
	Disseminated intravascular coagulation and purpura fulminans, Sneddon's syndrome (usually presents with livedo reticularis), cholesterol emboli
	Calciphylaxis
	Warfarin-induced necrosis (and heparin-induced necrosis), livedoid vasculopathy, Degos disease (malignant atrophic papulosis)

Adapted and translated from Morton & Phillips.^13^

a Most common causes of chronic ulcers in the legs.

## Venous ulcer

Venous ulcers (VU) occur in the most advanced stage of chronic venous disease.

### Clinical features that aid in diagnosis

Its main clinical features^3^, ^4^:

• Shape: Irregular, superficial in the beginning, and deepening as it evolves, with well-defined edges and commonly with yellowish exudate. The ulcer bed may have devitalized and colonized tissue; necrosis is rarely observed.
• Location: Distal portion of the legs (gaiter area), particularly on the medial malleolus region and rarely occurring on the upper calf and feet.
• Skin around the ulcer: Purpuric and hyperpigmented (ochre dermatitis); eczema can occur, evidenced by erythema, vesicles, flaking, pruritus, and exudate; varying degrees of induration and fibrosis indicate lipodermatosclerosis or fibrosing panniculitis, which can occur with or without ulcers; atrophic stellar scars of an ivory white color can be observed, with surrounding telangiectasias (atrophie blanche), located mainly on the distal third of the leg.
• Varicose veins and leg edema may be present.
• Pain: When present, it is of variable intensity; in general, it worsens at the end of the day with the orthostatic position and improves with limb elevation.
• Peripheral pulses: It is important to palpate the posterior tibial pulse and dorsalis pedis pulse; pulses should be present, but when decreased or absent, an association with arterial disease should be investigated.

### Complementary diagnosis

Objective tests may be necessary to confirm the diagnosis, determine the etiology, locate the anatomical site of the venous disease (superficial, deep, and perforating venous system) and the severity of the disease, or identify coexisting peripheral arterial disease.^5^

The main recommended complementary exams are:

a) Ankle-brachial index (ABI): it is important when there are doubts about the coexistence with arterial disease, *i.e*., reduced or absent peripheral leg pulses. It is the reason for the higher value of systolic blood pressure in the ankle when compared with the systolic blood pressure in the brachial artery. An ABI < 0.9 indicates arterial insufficiency component, influencing the onset of the ulcer. In elderly patients and/or with diabetes mellitus, when ABI > 1.2, the hallux/brachial index should be calculated; values > 0.6 suggest adequate arterial flow.
b) Duplex venous mapping: the non-invasive exam of choice to assess the superficial, deep, and perforating venous system; it allows functional assessment, *i.e*., to identify whether the venous disease is due to reflux, obstruction, or both.^6^

Phlebography, venous angiotomography, and venous angioresonance are indicated in specific cases, especially when duplex venous mapping is not conclusive.

### Treatment

The treatment of VU involves measures to eliminate or reduce the effects of venous hypertension (compression therapy, surgical treatment for venous abnormality), local treatment of the ulcer, systemic drugs that aid healing, and complementary measures.

#### Compressive therapies

**Figure 1:**
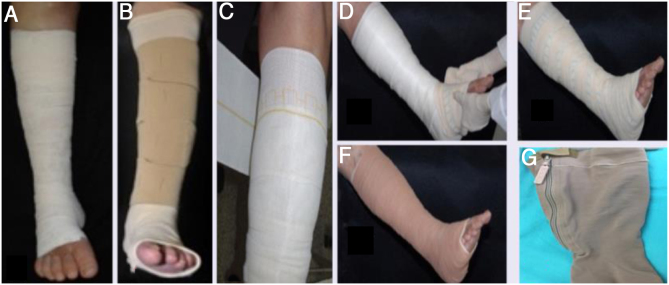
(A), Unna's boot; (B), velcro system; (C), elastic band (single layer); (D−F), multilayered compression; (G), high compression elastic stocking, with zipper.

They are the first treatment line for VU.^7^ They apply external pressure on the limb, which in turn improves venous hemodynamics. The external pressure that the compression must apply to the ankle is around 35 to 40 mmHg, and is gradually lower in the region below the knee. To achieve the benefits of compression, the patient must be encouraged to walk. The most widely used methods available are compressive bandages (Fig. 1A-F) and high-compression elastic stockings (Fig. 1G). High-compression stockings can be used if the ulcers are not very large. After the ulcers heal, compression stockings with a pressure of 30 to 35 mmHg are essential to prevent recurrence (Table 2).^4^

**Table 2:** Main characteristics of the compression methods used to treat venous ulcers.

**Inelastic compression bandages**
Provides high pressure during walking and small pressure at rest.
May remain in place for up to seven days, but presents changes in pressure over time.
Requires trained health staff for its placement.
Examples: Unna boot - Viscopaste®, Flexi-dress® (Fig. 1A); a variant is the Velcro system (Circaid® (Fig. 1B) that can be applied daily by a caregiver after adequate training.
**Single layer elastic compression bandages**
Greater stretch than inelastic strains and provides high pressure both while walking and resting (Fig. 1C).
May be applied by the patient or caregiver.
Examples: Surepress®, Tensopress®
**Multilayer compression elastic bandages (two to four layers)**
Provides sustained high pressure over time and can remain in place for up to seven days.
Requires a trained health staff for its placement.
Examples: Coban-2®, Dyna-flex® (first layer, for absorption, is composed of viscose fibers - Fig. 1D; second layer is the elastic compression bandage – Fig. 1E; third layer is an adhesive and cohesive compression bandage – Fig. 1F).
**High compression elastic stockings**
Ulcer kits: consists of two compression stockings, the first offers mild compression (15 to 20 mmHg) and is used to keep the dressing in place and remain overnight, while the second provides greater compression (20 to 32 mmHg) and must be placed on top of the previous one during the day (examples: Ulcer X Sigvaris®).
Zippered socks (Fig. 1G): to facilitate application, offering ankle pressure of 30 − 40 mmHg or 40 − 50 mmHg (examples: Ulcercomfort Venosan®, Ulcercare®).

Important information related to compression therapy:

a) According to a systematic review, the multilayer system is more effective than single layer systems.^8^
b) The choice of the method depends on several factors: availability of the resource, adaptation of the patient/caregiver, cost, and adverse events.
c) All compression methods are contraindicated if the patient has severe peripheral arterial disease.
d) The use of compressive therapy is limited by pain, excessive exudation, and difficulty in application.

#### Surgical treatment of venous abnormality

Although not in the scope of this consensus, it is important to comment that surgical correction of the underlying venous disease should be performed whenever possible, as surgery can promote healing, in addition to improving long-term prognosis due to the lower rate of VU recurrence.^9^

#### Local ulcer treatment

In addition to compressive therapy, local treatment includes cleansing, debridement techniques, and dressings that minimize infection/colonization and facilitate healing. These approaches are described in the topic “General principles for local management of chronic ulcers.”

It is important to note that in cases that do not respond to standard clinical treatment, skin autograft is an alternative. Although this therapy promotes healing in many cases, it is controversial in the literature as an exclusive measure, since the frequency of ulcer recurrence is high.^10^

### Systemic drugs that aid healing

Some systemic drugs can increase the rate of VU healing. The following are recommended as treatment adjuvants:

a) Drugs that affect the venous tone or phlebotonics: natural and synthetic flavonoids (diosmin – 1 g/day). A systematic review concluded that these drugs improve symptoms and edema related to chronic venous disease; however, no improvement in healing was observed.^11^
b) Drugs that affect blood flow properties (hemorheological agents): the best scientific evidence is in relation to pentoxifylline, for which a systematic review showed an effective adjuvant effect with compression therapy for the treatment of venous ulcers at a dose of 800 mg three times a day.^12^

### Complementary measures

a) Rest: Decreases the effects of venous hypertension. It should be performed with the leg elevated above the level of the heart, around three to four times a day, for at least 30 minutes.
b) Walking: Short walks, three to four times a day, should be stimulated, as they improve the action of the calf muscle pump.

## Arterial ulcers

Arterial disease is responsible for approximately 25% of leg ulcers.^13^ These lesions arise as a result of an inadequate arterial blood supply. Its most common cause is atherosclerotic disease, however thromboembolism can cause cutaneous infarction and lead to ulceration.^14^

Smoking, diabetes mellitus, advanced age, and history of arterial disease (both family and personal history in other locations) are considered risk factors.^14^

### Clinical features that aid in diagnosis

Symptoms of intermittent claudication, although typical of arterial disease, may go unnoticed due to a relative tendency to immobility in these patients. They are usually painful ulcers, even when small in diameter, with worsening pain when elevating the limb and some relief when placing it in a hanging position.^14^

These ulcers are usually located on the lateral or pre-tibial portions of the legs, as well as on the back of the feet or on bony prominences. Classically, they have a rounded shape, a well-demarcated border, a pale and sometimes necrotic bottom, and minimal or absent exudate.^13^, ^14^, ^15^ The extremities are cold, the capillary filling time is slow (> 3 − 4 seconds), and peripheral arterial pulses are very reduced or absent.^13^, ^16^

As a consequence of arterial hypoperfusion, trophic changes can be observed, such as pale, thin, scaly skin, with thinned hair and thickened nails.^14^, ^15^

### Complementary diagnosis

When resources are limited, the diagnosis of arterial disease by manual palpation of the pulses can be considered a reliable method.^17^ However, in diabetic patients, the presence of a palpable pulse does not completely rule out peripheral arterial disease.

ABI measurements are also considered valid as criteria for the severity of peripheral arterial disease. ABI < 0.9 indicates peripheral arterial disease, and values < 0.5 are associated with more advanced arterial involvement and with low probability of healing. If the ABI exceeds 1.2, this may reflect arterial calcification, which makes the arteries non-compressible; in these cases, the hallux/brachial index can be used.^18^

The measurement of transcutaneous oxygen tension (TcPO_2_) is a non-invasive method considered a good indicator of critical limb ischemia.^17^ This technique uses sensors placed on the area of interest; calluses, edema, and bony prominences should be avoided. The sensor heats the skin, causing hyperemia and facilitating the diffusion of oxygen. The measurement of PO_2_ in the dermis is obtained in mmHg; on the feet, values > 50 are considered normal. A value < 40 has been associated with hypoxia capable of compromising healing and values < 30, with critical ischemia. Currently, this method has even been suggested as a way to guide the choice of amputation levels.^19^

Arterial eco-Doppler is a little invasive and low-cost method used to confirm the diagnosis of arterial disease. Computerized angiography and magnetic resonance angiography are used in advanced peripheral arterial disease; they are important in the identification of the exact anatomical location of arterial occlusion and in the definition of revascularization techniques by the vascular surgeon.

### Specific management

The reduction of risk factors is recommended for all patients with arterial disease; it includes smoking cessation, reduction of serum lipids, and control of hypertension and diabetes, associated with antiplatelet therapy.^20^

Cilostazol is a vasodilator indicated for the treatment of intermittent claudication associated with early stages of peripheral vascular disease.^21^ The recommended dose is 100 mg orally twice daily; a reduction to 50 mg twice daily should be considered when there is concomitant administration of CYP3A4 inhibitors, such as diltiazem, erythromycin, ketoconazole, and itraconazole, as well as during coadministration of CYP2C19 inhibitors, such as omeprazole. It is generally well tolerated; however, common adverse effects include headache, diarrhea, and palpitations. Contraindications for use are class III or IV heart failure and a history of ischemic cardiomyopathy. It should be used with caution in patients with atrial fibrillation or flutter. It can also induce leukopenia, thrombocytopenia, and even agranulocytosis, reversible with discontinuation of the medication.^21^

The specific treatment of arterial ulcers is aimed at correcting the flow of arterial blood supply, which can be done through a surgical or pharmaceutical approach. In a review of the Cochrane database, updated in 2020, no evidence that topical agents or dressings can influence the healing of arterial ulcers was retrieved.^22^

In general, surgical debridement of arterial ulcers should be avoided, as it causes a greater demand for oxygen in the adjacent tissue, inducing hypoxia and potentially contributing to increased necrosis and wound size. Irreversible tissue loss (dry gangrene or eschar) must be left dry, as moisture can make the wound the ideal medium for bacterial growth. The vascular surgeon may opt for debridement at the time of revascularization, under appropriate antibiotic coverage.^20^

In the case of advanced stage arterial ulcers, the main therapeutic focus is on reducing pain and preserving the leg. The first line of treatment is revascularization, both by endovascular procedures and by open surgery. However, in one-third of the patients, revascularization procedures are not possible, have little chance of success, or are not effective. In such cases, prostaglandin E1 derivatives may be indicated.^23^

Hyperbaric oxygen therapy is an adjuvant treatment in patients who cannot undergo reconstruction or who did not present healing despite revascularization. A Cochrane review concluded that hyperbaric oxygen therapy significantly reduced the risk of amputations in diabetic patients, provided it was performed as part of an interdisciplinary wound care program.^24^

### Indications for emergency intervention

Attention to clinical and laboratory signs of infection should be increased in cases of patients with arterial disease. Signs such as increased ulcer size, temperature, exudate, erythema, odor, and edema, in addition to the appearance of new ulcers, indicate infection.^20^ If at least three of these signs are present, systemic antibiotic therapy should be indicated.

Peripheral arterial disease increases the risk of infection by multi-resistant bacteria and amputation, especially in elderly and diabetic patients.^25^ An increase in pain, as well as clinical worsening and signs of ischemia, may be an indication for surgical intervention (angioplasty or by-pass).^20^

## Neuropathic ulcers

The most prevalent causes of neuropathic ulcers (NU) are diabetes, leprosy, and alcoholic neuropathies.^26^ However, the differential diagnoses include syphilis, myelodysplasia, sarcoidosis, HIV and HTLV infection, hereditary disorders such as familial amyloidosis, and familial ulcerative-mutilating acropathy (Thevenard syndrome), among others.^27^, ^28^

### Clinical features that aid in diagnosis

Clinical manifestations of NU begin even before the ulcer itself is established. Disorders of autonomic nerves cause skin changes such as dry and thick skin and, as a consequence, fissures are observed in the plantar regions.^1^ The decrease in tactile, pain, and proprioceptive sensitivities, alterations in gait, and even paralysis in the most severe cases with involvement of motor nerves, lead to the formation of calluses, especially in the points of greatest support, which, if not treated correctly, can culminate in ulcers.^29^, ^30^

It is important to observe whether there are deformities in the feet, such as claw toes, changes in the arching, and signs of Charcot arthropathy.^30^ The physician should inquire about the time of onset of the manifestations, in addition to ruling out congenital and constitutional deformities.

It is essential to assess limb perfusion, as NU can be associated with ischemic ulcers, especially in diabetic patients. In exclusive neuropathic ulcers, the pulses are generally preserved and wide. The color of the feet is normal or even erythematous (due to an autonomic disorder), being distinct from the pale and cyanotic feet observed in patients with ischemia.^29^, ^31^

**Figure 2:**
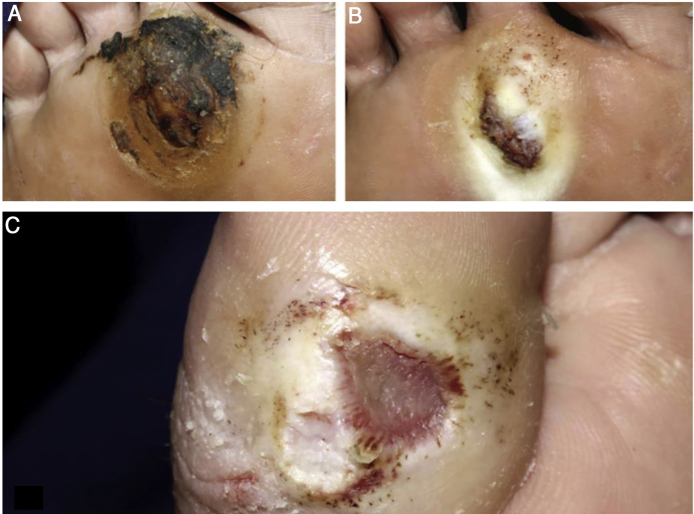
(A), hyperkeratosis and hematic crusts on the forefoot; (B), after cleaning, granular and hyperkeratotic ulcer; (C), ulcer with callous edges on the hallux.

**Figure 3:**
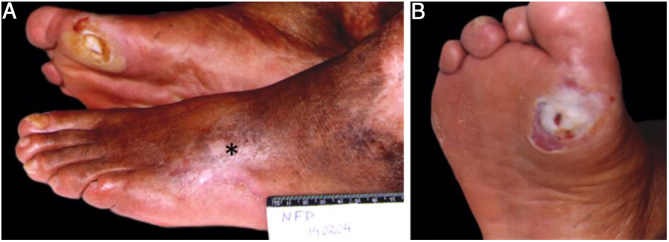
Diabetic patient: (A), scar of arterial ulcer (*) on the left foot and neuropathic ulcer over the first right metatarsal; (B), thinned peripheral calluses.

The ulcer is always preceded by non-painful calluses, which can develop purplish and/or blackened spots indicative of tissue distress and necrosis. The ulcer presents with a hyperkeratotic ring around it, forming a callous border; the center is deep and grainy. Hemorrhagic areas, indicating trauma or local friction, are observed.^32^ The most frequent location is the plantar region, mostly at the support points, equivalent to the foot triangle, generally on the forefoot at the first and fifth metatarsals and on the calcaneus, in addition to the plantar regions of the distal phalanges of the toes (Figure 2, Figure 3).

The main complications of NU are local skin infections and osteomyelitis.^32^, ^33^ The signs of infected NU are a foul odor, presence of yellow-greenish exudate, the appearance of local pain, and the presence of excess slough. Ulcers with necrosis are more prone to infection. In turn, osteomyelitis can be silent and manifest as an ulcer that is difficult to heal and exudate output, in addition to the presence of pain due to activation of deep periosteal nerve endings.^33^

There may be varying degrees of sensory, autonomic, and motor changes. The main complaints of patients are burning, tingling, sharp pain, limb edema, and loss of sensation. In more advanced cases, difficulty in walking, loss of shoes while walking, or even musculoskeletal deformities due to sequelae have been reported.^27^, ^32^, ^33^

### Complementary diagnosis

The main tools are anamnesis and physical examination.^30^ The evaluation of these ulcers depends on a thorough dermato-neurological examination, as well as on knowledge and application of the anatomy and physiology of peripheral nerves.

Symptom onset time should be characterized as acute (< 1 month), subacute (between 1 and 3 months) or chronic (> 3 months).^30^ The type of neuropathy also contributes to the investigation of the cause; it may be intradermal, mononeuropathy, multiple mononeuropathies, or polyneuropathy. In addition, the types of nerve fibers affected are of fundamental importance in the diagnosis: sensory (pain, temperature, vibration, tactile, and kinetic-postural), autonomic, and motor fibers.^26^, ^28^, ^30^

The main sensitivities that can be tested are: tactile, which is evaluated through Semmes-Weinstein nylon monofilaments (esthesiometer) and allows semi-quantitative assessment, essential for diagnosis and follow-up; pain, which can be tested with a needle and is more sensitive than the tactile sensitivity test;^29^, ^30^ thermal, which can be assessed using test tubes filled with hot and cold water; and the vibration sensitivity, using a 128 Hz tuning fork. All of these sensitivity tests should be performed symmetrically, in homologous regions.^30^

Trophic changes usually present as muscle hypotrophy. The neurological assessment may indicate the presence of hyporeflexia.^30^ Motor changes are graded through a manual strength test from 0 to 5, where 0 represents complete paralysis without moving the joint, 1 is the minimum degree of muscle contraction, and 5 represents maximum normal muscle strength.^34^

The most appropriate laboratory and complementary exams to assess neuropathies are those for the diagnosis/monitoring of diabetes.^33^ The assessment of neuropathy secondary to leprosy begins with the search for areas with altered sensitivity in the skin that may or may not coincide with hypochromic macules or erythematous-infiltrated plaques, and palpation of peripheral nerves that may show asymmetries and/or focal changes regarding thickening, pain, and shock. Peripheral nerve ultrasound can be used to demonstrate asymmetric or focal thickening, especially in the ulnar nerves,^35^ whereas in diabetes mellitus, nerve palpation is painless, with symmetrical and non-focal thickening.^35^, ^36^

Electroneuromiography is important for the differential diagnosis: a symmetrical and diffuse polyneuropathic pattern is observed in diabetes mellitus, while a pattern of multiple asymmetric and focal mononeuropathy is observed in leprosy. Such exams contribute to the diagnosis and monitoring of the neuropathy in question.^37^

### Specific management

Treatment should start with prevention. This includes daily inspection of the feet, cleaning and drying of the interdigits, straight nail cutting, hydration, lubrication (avoiding the interdigits), foot massage, restriction to walking barefoot, monitoring sensitivity with monofilaments, removing calluses, and examination of the pulses and of the presence of deformities of the feet.^38^, ^39^ Physicians should recommend the use of shoes without internal seams, made of comfortable fabrics and usually a size larger, in addition to seam-free socks, in order to avoid any possibility of pressure points.

In patients with neuropathy already installed, the approach must be multidisciplinary; the treatment of the underlying disease is important, as well as the evaluation of shoes and orthoses/insoles to avoid repetitive local trauma.^38^ Diabetes patients must maintain strict glycemic control; leprosy patients must undergo the usual and indicated treatment, in addition to treating reactions early.^30^ Patients with alcoholic neuropathy or other hypovitaminosis should receive adequate vitamin replacement and cease the habits that cause the deficiencies.^33^, ^38^

Physiotherapeutic support is important for the early detection of risk areas, with functional assessment for early diagnosis of sensory and motor loss,^40^ which contributes to the promotion of the patient’s health, with muscle strengthening, balance, and proprioception practices aiming at improving and maintaining the sensitivity and musculature of the foot.^40^, ^41^ The indication of insoles, orthoses, and shoes should be individualized, especially when the foot already has amputation scars, which changes its biomechanics, predisposing it to a greater risk of ulcerations and new amputations.^42^ Manual and electrothermal phototherapy techniques also contribute to wound healing and scarring improvement.^42^, ^43^

The initial specific treatment of the NU is to remove the calluses by eliminating this “natural” pressure point, both from the center and in the peripheries of the already open ulcer.^42^, ^43^, ^44^ After this approach, debridement products (collagenases and fibrinases, among others) are used if there is still devitalized tissue; otherwise, dressings with calcium alginate in the center are used, covered with hydrocolloid plaque. Both are used in association with secondary dressings that offer reduced pressure points, in addition to insoles and suitable footwear. Changes of dressings vary depending on the product used, from two times a day with collagenase, to every three days with calcium alginate and hydrocolloid. However, the site should be inspected daily, due to the risk of infectious complications; in these patients, in general, the classic phlogistic signals do not appear early due to systemic and local metabolic disorders.

Although NU are generally painless, they can be associated with neuropathic pain, as in cases of chronic decompensated diabetes mellitus and in leprosy, usually after treatment, with recurrent reactional neuritis.^8^ For these patients, first-line treatment is amitriptyline at doses of 25 to 75 mg/day or nortriptyline at doses of 25 to 150 mg/day; the latter presents a better safety profile. Other options include duloxetine at doses of 30 to 120 mg/day and venlafaxine at doses of 150 to 225 mg/day.^28^

Also as a first-line treatment, gabapentin (up to 2,400 mg/day) and pregabalin (up to 600 mg/day) are considered effective in diabetic neuropathy.^28^

For second-line treatment, tramadol and opioids can be used. In acute neuropathic pain or flares, such medications can be used as the first line. The maximum dose of tramadol is 400 mg/day. Patients with a personal or family history of drug abuse should be counseled and are more likely to misuse such medications.^28^

As a third-line treatment, citalopram at doses of 40 mg/day (in the elderly, the maximum dose should be 20 mg/day) and paroxetine 60 mg/day (in the elderly, the maximum dose should be 40 mg/day) can be used.^28^ Carbamazepine at doses of 200 to 600 mg/day, lamotrigine 400 mg/day, and valproate up to 1,200 mg/day are other options described in the literature.^28^

## Martorell's hypertensive ulcer (MHU)

Martorell's hypertensive ulcer (MHU) is a less common and probably underdiagnosed cause of chronic ulcers of the legs.^45^, ^46^, ^47^

### Clinical features that aid in diagnosis

It is clinically characterized by a necrotic, phagedenic ulcer with an erythematous halo. It has a rapid growth and is extremely painful, with symptoms disproportional to the size of the ulcer.^19^, ^45^, ^47^ Severe pain does not improve with elevation of the limb or rest. The most common location is the anterolateral distal region of the leg,^45^, ^50^, ^51^ followed by the Achilles tendon region (about 15% of cases).^46^ Ulcers can be bilateral in approximately half of the cases, and satellite lesions are common.^45^, ^46^, ^50^ By definition, patients have a long-standing and poorly controlled history of systemic arterial hypertension (SAH).^49^ Type 2 diabetes mellitus is the most prevalent associated comorbidity, observed in up to 60% of cases.^45^, ^46^, ^47^ A history of previous trauma at the site of the ulcer onset is reported by half of the patients.^45^

MHU has been described in patients without signs of arterial or venous insufficiency,^45^, ^49^, ^51^ and patients classically have normal ABI.^45^, ^47^ However, some authors report a concomitant occurrence of peripheral arterial insufficiency in approximately 50% of patients,^46^, ^47^ in addition to other conditions, such as obesity.^48^, ^52^

### Complementary diagnosis

The diagnosis of MHU is based on clinical characteristics, histology, and exclusion of differential diagnoses.^53^ Arterial and venous ulcers can be distinguished by clinical and complementary imaging tests.

Surgical biopsy should always be performed, preferably extending to the fascia.^47^, ^50^ Punch biopsy can lead to an incorrect diagnosis and should be avoided.^46^ The most common histopathological findings are arteriosclerosis in the subcutaneous tissue, calcinosis, and occasionally hyalinosis of the middle layer of the arterioles and intimal hyperplasia.^45^, ^46^, ^50^, ^51^ These findings are not pathognomonic and can occur in calciphylaxis. Therefore, all patients should be screened for chronic kidney disease and alterations in phosphorus and calcium metabolism.^45^, ^46^, ^50^, ^53^

Due to its rapid growth and erythematous border, MHU is often confused with pyoderma gangrenosum. In addition to inadequate treatment with immunosuppressants, this incorrect diagnosis delays the onset of debridement and other surgical treatment modalities. Clinical diagnosis associated with histology is fundamental to distinguish between the two entities, since pyoderma gangrenosum frequently presents as an ulcer with violet undermined borders and, on histopathological examination, an abundant neutrophilic inflammatory infiltrate is observed.^45^, ^46^, ^47^, ^50^, ^53^, ^54^

### Treatment

The treatment of MHU can be divided into systemic therapy, pain control, surgical treatment, conservative treatment, and preventive measures.^45^ Adequate treatment of SAH is always indicated, but it is not able to reverse the established tissue changes and act effectively in healing the ulcer.^46^, ^53^ Non-selective beta-blockers are contraindicated.^45^ The use of oral anticoagulants such as vitamin K inhibitors and antiplatelet agents is indicated.^45^, ^53^

Pain control is always challenging in the face of the excruciating pain referred by patients. Common painkillers and opioids should be used.^45^ However, surgical treatment of MHU is considered the first line for pain control.^49^

Surgical debridement, followed by partial skin grafts, promotes important pain relief and accelerates healing. It is the most effective treatment for ulcers over 3 cm in diameter.^47^, ^49^ The mean post-surgical healing period is two weeks, *vs*. 15 months when opting for conservative treatment.^49^ About half of the patients need only one procedure, while others may need two or three successive grafts.^50^, ^53^ It is worth mentioning that all series of cases treated with skin graft were performed in patients with MHU without associated arterial or venous disease. The literature does not feature data to allow an estimation of the success rate of treatment in patients with mixed ulcers. Negative pressure therapy can be performed after grafting in an attempt to increase the effectiveness of the procedure.^45^, ^53^

In patients with chronic pain that is refractory to the therapies described above, electrical stimulation of the spinal cord and lumbar sympathectomy are described as possible therapeutic modalities, but with variable results and potentially serious side effects.^46^, ^47^, ^51^

Conservative treatment aims to remove devitalized tissues, maintain adequate humidity, and control bacterial load. It is the therapeutic modality of choice in patients with small ulcers (< 3 cm) and in ulcers that are growing.^47^ Compressive therapy (25 − 30 mmHg) should be instituted whenever the pain is already under control and there is no contraindication detected by ABI.^45^

Preventive treatment includes optimization of antihypertensive therapy, smoking cessation, skin care (such as hydration), and prevention of local trauma.^45^

## General principles for local management of chronic ulcers

Regardless of the cause of the chronic ulcer, local management must be based on the knowledge of some principles, such as the TIME concept, cleaning, debridement, dressings, and biofilm control.

### TIME concept

Due to the difficulty in healing a chronic ulcer, a systematic approach focused on correcting imbalances became necessary.^55^ Table 3 describes the clinical observations and interventions related to the preparation of the ulcer bed, based on the acronym TIME (Tissue, Infection/Inflammation, Moisture, and Edge).^56^, ^57^, ^58^

**Table 3:** TIME concept.

	Cause	Management	Results
T: *Tissue* – Non-viable or deficient tissue	Defective ECM	Debridement (autolytic, surgical, enzymatic, biological, or mechanical)	Restoration of the base of the wound and ECM functional proteins
	Cell debris altering healing		
		Negative pressure therapy	
Purpose of conduct: Viable wound base			
I: *Infection/Inflammation*	Biofilm	Biofilm control: antiseptic,^a^ systemic antimicrobials^b^	Low bacterial count
	Prolonged inflammation		Controlled inflammation
	Inflammatory cytokines		Cytokines
	High protease activity		Low protease activity
	Low growth factor activity		High growth factor activity
Purpose of conduct: Bacterial balance and inflammation control			
M: Moisture – changes in the exudate	Lack of exudate: slows down cell migration	Hydration with hydrogel or hydrocolloid	Cell migration restored by moisture balance
	Excess: maceration of the edges	Alginate/hydrofiber/foam	Control of excess liquid
		Compression therapy	Maceration prevented
		Negative pressure therapy	
Purpose of conduct: Moisture balance			
E: *Edge*	No migration of keratinocytes from the edges	Reassess cause or consider auxiliary therapies: Debridement; grafts; biological agents; pharmacological therapies; other technologies^c^	Keratinocyte migration
	ECM abnormalities		Responsive cells
	Abnormal protease activity		Restoration of the appropriate protease profile
Purpose of conduct: Advance the wound edge			

ECM, extracellular matrix.

a See antiseptics in dressings.

b Indication of systemic antibiotic therapy (see text).

c Ultrasound, electromagnetic therapy, low-level laser therapy, negative pressure therapy.

### Cleaning of chronic ulcers

Devitalized tissues, foreign bodies, and debris are factors that prevent the healing of wounds; its removal is essential for healing. Cleaning is the initial step in treatment and should be carried out by applying non-toxic products without damaging existing viable tissue. Chronic ulcers can be cleaned with saline solution, tap water, and polyhexanide (polyhexamethylene biguanide [PHMB]); the latter is an important tool, but its cost is high.^59^

a) 0.9% saline solution: Suitable due to being isotonic, having the same pH as plasma, being hypoallergenic, and not interfering with the healing process. However, it is insufficient to clean colonized/infected tissue.
b) Tap water: Easy to access, but not superior to saline solution.^60^
c) PHMB: Good bactericidal efficacy; low-risk antiseptic with excellent tolerance profile, low risk of contact sensitization, and antimicrobial action in infected acute or chronic lesions. Its physical-chemical action prevents the onset of bacterial resistance.^61^ When associated with the surfactant betaine, it produces an autolytic debridement effect.

Povidone-iodine is a known antiseptic, but its use in the treatment or prevention of infection in ulcers is debatable, as it can cause allergy, has low penetration, and has a toxic effect on cells, disturbing tissue regeneration. However, a systematic review of randomized clinical trials^62^ showed that iodine did not prolong or reduce the healing time of chronic ulcers when compared with other antiseptics or dressings.

Solutions such as chlorhexidine, acetic acid, potassium permanganate, and Dakin's solution, although safe on intact skin, can be toxic to granulation tissue, by prolonging the acute inflammatory response and delaying collagen production; therefore, they are not recommended for chronic ulcers.

### Debridement methods

Tissue abnormalities in chronic wounds trigger the accumulation of devitalized and necrotic tissues; regular debridement is necessary to reduce necrosis and achieve healthy granulation tissue.^63^, ^64^

Regular debridement helps healing by removing the biofilm, improving the biodistribution of antimicrobials, and preventing the formation of a new biofilm.^65^

Debridement, by definition, is any method of removing devitalized, necrotic, infected, fibrinous, or foreign material from a wound.^66^ The main methods are:

a) Surgical:
- Proper surgical debridement: Performed in a surgical environment under anesthesia and indicated for large areas of devitalized and/or necrotic tissue or extensive cellulitis, infected bone, or sepsis not responsive to other techniques; a rapid technique with inherent risks of bleeding and transient bacteremia, in addition to damage to structures such as nerves and tendons; requires trained medical personnel and has a higher cost.
- Conservative surgical debridement: Performed at the bedside, it aims to remove devitalized tissue or foreign material inside or around the wound, under local anesthesia using a scalpel, scissors, or curette. This technique is considered less aggressive and less selective when compared with the surgical technique; however, it is faster, and has similar risks of pain and bleeding in the postoperative period. Anesthesia (topical or injectable) is recommended to minimize discomfort.
b) Mechanical:
- Wet-to-dry: Application of gauze moistened with saline, which is left to dry and adhere to the bed of the wound, and subsequently removed by traction, removing the devitalized tissue with it. It is considered to be a low-cost, slow, non-selective, and painful method.
- Hydrotherapy: Water pressure is used in the form of a jet, directed to the surface of the lesion to remove devitalized tissue. Special care is required for ischemic ulcers.
c) Autolytic: Highly selective technique, performed using hydrogel and hydrocolloid dressings. It acts by retaining exudate from the wound by forming a collection of endogenous proteolytic enzymes produced by macrophages (such as collagenases, elastases, myeloperoxidases, and metalloproteinases) whose purpose is to liquefy and separate devitalized tissues from healthy tissue. This technique can cause perilesional maceration, requires minimal clinical training, is painless, and has a slow response. In order to be applied, there must be a minimum of exudate. Contraindicated in critically colonized, infected, and ischemic ulcers.
d) Enzymatic: It uses a topical substance containing an exogenous enzyme capable of digesting devitalized tissue. This technique requires daily changes. In Brazil, three products are available: papain, collagenase, and fibrinolysin/DNase (Table 4).^67^, ^68^, ^69^, ^70^, ^71^

**Table 4:** Characteristics of topical therapies with debridement action.

Types	Indications	Contraindications	Instructions for use
Papain:^68^ Proteolytic enzymes of papaya; *in vitro* antibacterial action; stimulates granulation; non-selective enzymatic debridement	Any type of ulcers: with or without biofilm; with variable volume of exudate; 2% to 4% lesions in the granulation phase with variable exudate; 6% lesions with necrosis	Hypersensitivity	Daily exchange
		Maceration	Local application with gauze soaked in the solution
		Do not place on fascia/cartilage/tendon/bone	
Collagenase:^69^ Formed by clostridiopeptidase A; it may or may not contain chloramphenicol; selectively degrades native wound collagen	Safe selective enzymatic debridement on fascia/cartilage/tendon/bone; any type of ulcers	Healing by first intention	Daily change
		Allergy	Apply thin layer and cover with secondary dressing
Fibrinolysin:^70^ fibrinolysin, deoxyribonuclease, and chloramphenicol; enzymatic debridement	Safe selective enzymatic debridement on fascia/cartilage/tendon/bone; any type of ulcers	Healing by first intention	Daily change
		Allergy	Apply thin layer and cover with secondary dressing
e) Biological (larvae): Uses *Lucilia sericata* larva; however, few studies have proved its effectiveness for VU debridement.

The most used methods in clinical practice are surgical, enzymatic, and autolytic debridement.

### Biofilm control

Biofilms are structured communities of microbial cell colonies wrapped in a polymeric matrix and adhered to surfaces (natural or artificial) or to themselves. They are heterogeneous and dynamic, maintain variable genetic diversity and gene expression (phenotype), and are capable of creating environments and defenses that can produce chronic inflammation and delay healing. These colonies develop and protect themselves through the production of extracellular polymeric substances that give them structural integrity and protect them against external agents and, therefore, are characterized by resistance to antimicrobial and antiseptic agents, as well as resistance to the host’s immune system defenses. They are commonly composed of several species of microbial agents such as bacteria, fungi, and viruses.^72^

There is growing evidence that biofilms are present in most, if not all, chronic unhealed ulcers. In a recent meta-analysis of *in vivo* studies, it is highlighted that at least 78% of chronic ulcers contain them.^73^

It is essential to differentiate ulcers having only critical colonization or superficial infection (with biofilm), from those with a deeper infection leading to erysipelas, cellulitis, or lymphangitis.^74^ These are signs and symptoms that indicate infection of the tissue adjacent to the ulcer and require the introduction of systemic antibiotics: general signs such as malaise and loss of appetite, and local signs such as increased exudate, delayed healing, swelling at the base of the wound, persistent pain, friable granulation tissue, discoloration of the wound bed, foci of abscess, and fetid odor. Increased pain and wound size are probably the two most useful predictors.^58^

Bacteriological tests using swabs are not indicated to make this differentiation, as they qualitatively identify the presence of bacteria, but they cannot determine the quantity of bacteria, so they fail to differentiate simply colonized ulcers from those with deep infection. In cases of associated infection, when it is necessary to identify the bacteria to direct the treatment, biopsies of the ulcer base should be performed for culture after exhaustive washing of the bed with 0.9% saline.^75^

Suggested measures for biofilm control^76^:

a) Debridement: It is one of the most important strategies in the treatment of biofilms, but it does not remove them completely and, therefore, cannot be used alone.
b) Antiseptic agents, washing, or therapeutic irrigation should be used together with debridement methods to promote bacterial reduction and suppress their re-development.^77^ When considering topical antiseptics, those with antibiofilm properties and with less ability to induce cytotoxicity in healthy tissue are preferred. It is important to note that topical antibiotics such as neomycin, bacitracin, mupirocin, and silver sulfadiazine are not recommended for the treatment of chronic ulcers.
c) Topical antibiofilm therapies are described in Table 5, with emphasis on iodine alkoxide, PHMB, and silver-containing dressings.^78^, ^79^

**Table 5:** Characteristics of topical therapies recommended for combating biofilm.

Types	Indications	Contraindications	Instructions for use
9% cadexomer iodine 78, 79: Contains 0.9% iodine; antimicrobial action breaking the lipid membrane and inhibiting bacterial protein synthesis; non-toxic to fibroblasts; the iodine is being released as the exudate is absorbed	High capacity to absorb exudate; each gram absorbs 3 mL of the exudate; total/partial thickness; critically colonized/infected lesions; MSRA^a^	Iodine allergy Hashimoto thyroiditis Graves' disease	Dressing exchange every 2 to 3 days Exchange when the brown color turns yellow/gray
Biguanide polyhexamethylene^79^: Broad bacterial spectrum; its positive structure binds to the negative charges of the cell membrane, breaking its integrity; active in the biofilm; antiseptic; non toxic; non-irritating; active against MSRA,^a^ VRE,^b^ and fungi	Reduces bacterial biofilm	None	Variable depending on the protocol
Dressings and topical drugs containing silver^79^: antiseptic, anti-inflammatory and broad antibacterial action; blocks bacterial cell respiration; destroys bacterial membranes; denatures bacterial RNA and DNA; active against MSRA,^a^ VRE,^b^ and fungi	Reduces bacterial biofilm	Allergy to silver Use with caution in diabetic ulcers, due to the cytotoxic effect on fibroblasts	The silver ion release method varies depending on the product The dressing exchange is variable depending on the protocol

a Methicillin-resistant *Staphylococcus aureus*.

b Vancomycin-resistant enterococci.
d) Antibiofilm strategies should be used until the wound bed is visibly clean, presenting healthy granulation tissue and/or on the way to healing.
e) Systemic antibiotics are not able to eradicate biofilm from a wound; therefore, their use should be considered with caution aiming at attacking planktonic (surface) bacteria, acute infection, and prevention of associated systemic infections.

### Dressings and other technologies

**Figure 4:**
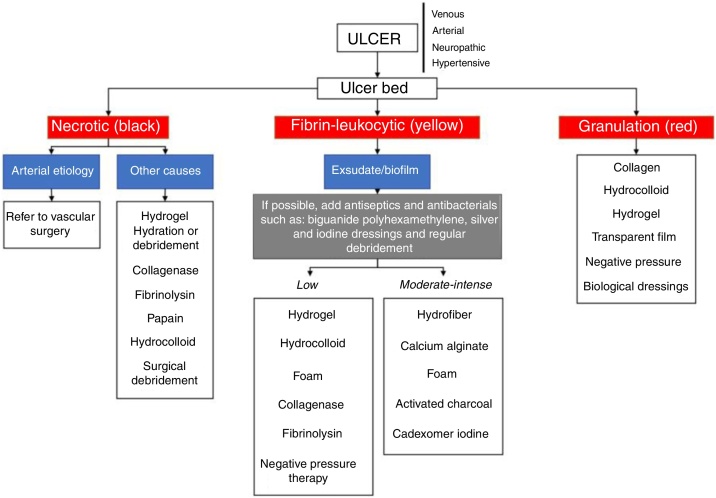
Flowchart with the main therapeutic measures according to the characteristics of chronic skin ulcers.

Table 6 and the flowchart of fig. 4 present information about the main dressings, such as foams, hydrogels, calcium alginate, activated charcoal/silver coverings, hydrocolloids, transparent films, Unna boots, hydrofiber, and collagen. Their indications are also described and should be used according to the characteristics of the ulcers.^79^, ^80^, ^81^, ^82^, ^83^, ^84^, ^85^, ^86^

**Table 6:** Characteristics of the main dressings.

Types	Indications	Contraindications	Instructions for use
Foam^79^, ^80^: Polyurethane; absorption of exudate; Maintenance of humid environment; Hydrophilic properties; Painless/atraumatic exchange	Total/partial thickness Moderate/high exudate Reduces pressure and friction Protects friable peri-ulcer skin	Third-degree burns Ischemic ulcers with eschar at the base Dry/necrotic bed Ulcers with fistulas	Primary/secondary dressing^a^ Depends on the volume of exudate (1 to 7 days)
Hydrogel^79^, ^81^: Hydrophilic cross-linked polymers with 80%−90% water; non-adherent; autolytic debridement; absorbs minimal amounts of exudate	Total/partial thickness	Third-degree burns	Apply at the base of the ulcer and cover with secondary dressing
	Dry lesions with minimal exudate	Moderate/high exudate	
		Ulcers with critical colonization and infection	Daily use
			Do not use as a wound filler
Calcium alginate^79^, ^82^: Derived from seaweed; exudate absorption; formation of humid environment by gel formation (calcium and sodium salts + exudate); hemostatic properties; autolytic debridement; non-stick due to gel formation	Moderate/high level of exudate Total/partial thickness Applied to surfaces and cavities	Dry/necrotic bed Third-degree burns Minimal exudate	Fix with secondary dressing When the dressing is saturated (1 to 7 days)
Activated charcoal/silver^82^: Double layer of fibers; external: coal; internal: silver; absorption of exudate; coal: adsorption of microorganisms; silver: bactericidal action	Chronic infected and exudative ulcers	Clean, granulating, and uninfected ulcers Do not use on fascia/tendons/bones	Some charcoal dressings can not be cut Fix with secondary dressing Exchange depends on saturation (1 to 7 days)
Hydrocolloids^79^, ^83^: It contains an internal self-adhesive layer and a gel-forming agent such as gelatin or carboxymethylcellulose (CMC); inner layer of hydrocolloid in foam or film; increases its thickness in contact with exudate; autolytic debridement; puts up a form barrier against pathogens	Total/partial thickness Minimum to moderate level of exudate Wound bed with granulation and necrosis	Third-degree burns Infection Bedsores	Direct application Adhesive edge 2.5 to 5 cm safety edge Exchange depends on exudate level (3 to 5 days) No secondary dressing required
Transparent films^84^: CMC in film form; aseptic; creation of a humid environment; exudate retention	Total/partial thickness Secondary dressing Abrasions/graft donor areas Minimum to moderate level of exudate	High level of exudate	Minimum safety edge 2.5 cm
Unna's boot^85^: 10% zinc oxide + starch on bandage of cotton fabric; non-elastic compressive action; stabilization of hydrostatic pressure; increased resistance to infections; creation of humid environment	Venous ulcers	Ulcers of ischemic origin	Prior preparation includes Trendelenburg position for 6 to 8 hours to reduce swelling Wrap the ankle/knee limb
Hydrofiber^79^, ^83^: Fiber CMC with or without silver; absorption of very much exudate; it forms hydrophilic gelatinous substance; provides humid environment; non-adherent; facilitates autolytic debridement, granulation and epithelialization	Total/partial thickness Moderate/high exudate	Dry or low exudate volume lesions	Fix with secondary dressing Exchange according to saturation (1 to 2 days)
Collagen^79^, ^86^: Bovine, porcine or sheep source; available in gel, tapes or powder; bioabsorbable; chemotactic for the cells involved; inactivates MMP, elastase and decreases the level of inflammatory mediators; stimulates endogenous collagen; *in vitro* bacteriostatic properties; some versions impregnated with silver; increases the epithelialization rate	Total/partial thickness Minimum/moderate volume of exudate Uninfected lesion	Allergy to source tissue	Apply directly to the ulcer bed and cover with secondary dressing Exchange depends on exudate level

Primary dressing is the first in direct contact with the ulcer bed; secondary dressing, transparent films or gauze-like fabrics or crepe stripe; MMP, metalloproteinases.

Other promising treatments, such as electrical stimulation, negative pressure therapy, hyperbaric oxygen therapy, ultrasound, and low-level laser therapy, have been used as adjuvants in the treatment of chronic ulcers; nonetheless, their respective systematic reviews indicate that further studies are necessary to certify their effectiveness.^24^, ^87^, ^88^, ^89^, ^90^

## Final considerations

Venous, arterial, neuropathic, and hypertensive ulcers are frequent, with an especially higher prevalence in the elderly population. The correct diagnosis of these conditions and adequate treatment, based on the best scientific evidence, are essential to reduce the negative social, economic, and quality of life impacts in affected patients.

Table 7 summarizes the main causes of chronic leg ulcers, with key points in diagnosis and treatment.

**Table 7:** Key points in the diagnosis and treatment of the main causes of chronic cutaneous leg ulcers.

Ulcer etiology	Diagnosis	Clinical treatment
Venous	Clinical: distal leg ulcer, edema, ochre dermatitis, lipodermatosclerosis, and varicose veins	Compressive therapy (high compression bands and elastic stockings)
	Distal pulses present (posterior tibial and pedal)	Cleaning, debridement, and dressings
	ABI between 0.9 and 1.2	Hemorheological and phlebotonic drugs: pentoxifylline and diosmin
	Duplex venous mapping: evaluates the superficial, perforating, and deep venous system	Anti-stasis measures: rest with raised limbs, walking
Arterial	Clinical: very painful ulcers, especially with elevated leg, in patients with a history of claudication	Control of risk factors (smoking, reduction of serum lipids, control of SAH, DM)
	Absent peripheral pulses	Platelet antiaggregant
	ABI < 0.9 (values < 0.5 indicate more advanced arterial disease)	Cilostazol
	TcPO_2_ < 40 (hypoxia capable of compromising healing) and < 30 (critical ischemia)	Cleaning and dressings (avoid surgical debridement)
	Arterial Doppler ultrasound	Oxygen therapy
Neuropathic	Clinical: ulcer in patients with a risk factor for neuropathy (*e.g*.: DM, leprosy and alcoholism), preferably in the plantar region and with callous edges	Control of the underlying disease (DM, leprosy, and alcoholism)
		Decrease plantar load: shoes and orthoses to prevent repetitive trauma
		Physiotherapeutic support
	Normal, decreased or absent distal pulses (the latter when associated with arterial disease)	Electrothermal phototherapy
		Trimming of the callous edges of ulcers and debridement agents
Hypertensive	Clinical: necrotic and painful ulcers in patients with severe and poorly controlled SAH	Control of SAH (contraindication for the use of non-selective beta-blockers)
		Oral anticoagulants: vitamin K inhibitors and antiplatelet agents
	Peripheral pulses present	
	Normal ABI (> 0.9)	Pain control: analgesics (opioids)
	Histopathological findings: arteriosclerosis and calcinosis	Surgical debridement and skin grafts
	Exclude calciphylaxis: screened for chronic kidney disease and changes in phosphorus and calcium metabolism	Cleaning and dressings

ABI, ankle-brachial index; TcPO_2_, transcutaneous oxygen tension; SAH, systemic arterial hypertension; DM, diabetes mellitus.

This consensus addressed the diagnostic and therapeutic management of chronic leg ulcers of the most common causes, based on scientific evidence and the experience of specialists, to assist dermatologists and other health professionals, in order to benefit the greatest number of patients with this condition.

## Financial support

None declared.

## Authors’ contributions

Luciana Patricia Fernandes Abbade: Elaboration and writing of the manuscript, collection, analysis, and interpretation of data.

Marco Andrey Cipriani Frade: Elaboration and writing of the manuscript, collection, analysis, and interpretation of data.

José Roberto Pereira Pegas: Elaboration and writing of the manuscript, collection, analysis, and interpretation of data.

Paula Dadalti-Granja: Elaboration and writing of the manuscript, collection, analysis, and interpretation of data.

Lucas Campos Garcia: Elaboration and writing of the manuscript, collection, analysis, and interpretation of data.

Roberto Bueno Filho: Elaboration and writing of the manuscript, collection, analysis, and interpretation of data.

Carlos Eduardo Fonseca Parenti: Elaboration and writing of the manuscript, collection, analysis, and interpretation of data.

## Conflicts of interest

None declared.

## References

[1] Agale SV (2013). Chronic Leg Ulcers: Epidemiology, Aetiopathogenesis and Management. Hindawi Publ Corp Ulcers..

[2] Green J., Jester R., McKinley R., Pooler A. (2014). The impact of chronic venous leg ulcers: asystematic review. J Wound Care..

[3] Abbade L.P., Lastória S., Rollo H.A. (2011). Venous ulcer: Clinical characteristics and risk factors. Int J Dermatol..

[4] Kirsner RS, Vivas AC (2015). Lower-extremity ulcers: diagnosis and management. Br J Dermatol..

[5] Vivas A, Lev-Tov H, Kirsner RS (2016). Venous Leg Ulcers. Ann Intern Med..

[6] Coleridge-Smith P., Labropoulos N., Partsch H., Myers K., Nicolaides A., Cavezzi A. (2006). Duplex ultrasound investigation of the veins in chronic venous disease of the lower Limbs − UIP consensus document. Part I. Basic principles. Eur J Vasc Endovasc Surg..

[7] Mościcka P., Szewczyk M.T., Cwajda-Białasik J., Jawień A. (2019). The role of compression therapy in the treatment of venous leg ulcers. Adv Clin Exp Med..

[8] O’Meara S., Cullum N., Nelson E.A., Dumville J.C. (2012). Compression for venous leg ulcers. Cochrane Database Syst Rev..

[9] Gohel M.S., Heatley F., Liu X., Bradbury A., Bulbulia R., Cullum N. (2018). A randomized trial of early endovenous ablation in venous ulceration. N Engl J Med..

[10] Jones J.E., Nelson E.A., Al-Hity A. (2013). Skin grafting for venous leg ulcers. Cochrane database Syst Rev..

[11] Martinez-Zapata M.J., Vernooij R.W., Uriona Tuma S.M., Stein A.T., Moreno R.M., Vargas E. (2016). Phlebotonics for venous insufficiency. Cochrane database Syst Rev..

[12] Jull A.B., Arroll B., Parag V., Waters J. (2012). Pentoxifylline for treating venous leg ulcers. Cochrane database Syst Rev..

[13] Morton LM, Phillips TJ (2016). Wound healing and treating wounds Differential diagnosis and evaluation of chronic wounds. J Am Acad Dermatol..

[14] Fonder M.A., Lazarus G.S., Cowan D.A., Aronson-Cook B., Kohli A.R., Mamelak A.J. (2008). Treating the chronic wound: A practical approach to the care of nonhealing wounds and wound care dressings. J Am Acad Dermatol..

[15] Star A (2018). Differentiating Lower Extremity Wounds: Arterial, Venous. Neurotrophic. Semin Intervent Radiol..

[16] Hafner J., Schaad I., Schneider E., Seifert B., Burg G., Cassina P.C. (2000). Leg ulcers in peripheral arterial disease (arterial leg ulcers): impaired wound healing above the threshold of chronic critical limb ischemia. J Am Acad Dermatol..

[17] Federman D.G., Ladiiznski B., Dardik A., Kelly M., Shapshak D., Ueno C.M. (2016). Wound healing society 2014 update on guidelines for arterial ulcers. Wound Repair Regen..

[18] Donohue C., Adler J.V., Bolton L.L. (2020). Peripheral arterial disease screening and diagnostic practice: A scoping review. Int Wound J..

[19] Arsenault K.A., Al-Otaibi A., Devereaux P.J., Thorlund K., Tittley J.G., Whitlock R.P. (2012). The Use of Transcutaneous Oximetry to Predict Healing Complications of Lower Limb Amputations: A Systematic Review and Meta-analysis. Eur J Vasc Endovasc Surg..

[20] Weir GR, Smart H, van Marle J, Cronje FJ, Sibbald RG (2014). Arterial Disease Ulcers, part 2: treatment. Adv Skin Wound Care.

[21] Balinski AM, Preuss CV (2019). Cilostasol.

[22] Broderick C., Pagnamenta F., Forster R. (2020). Dressings and topical agents for arterial leg ulcers. Cochrane database Syst Rev..

[23] Lawall H., Pokrovsky A., Checinski P., Ratushnyuk A., Hamm G., Randerath O. (2017). Efficacy and safety of alprostadil in patients with peripheral arterial occlusive disease fontaine stage IV: results of a placebo controlled randomised multicentre trial (ESPECIAL). Eur J Vasc Endovasc Surg..

[24] Kranke P., Bennett M.H., Martyn-St James M., Schnabel A., Debus S.E., Weibel S. (2015). Hyperbaric oxygen therapy for chronic wounds. Cochrane database Syst Rev..

[25] Aysert Yildiz P., Özdil T., Dizbay M., Güzel Tunçcan Ö, Hizel K. (2018). Peripheral arterial disease increases the risk of multidrug-resistant bacteria and amputation in diabetic foot infections. Turkish J Med Sci..

[26] Frade MA, Ferriolli E, Moriguti JC, Costa Lima NK (2012). Úlceras cutâneas. Desafios do diagnóstico diferencial em geriatria.

[27] Shai A, Maibach HI (2005). Wound healing and ulcers of the skin: Diagnosis and Therapy - The Pratical Approach.

[28] Kraychete DC, Sakata RK (2011). Painful Peripheral Neuropathies. Brazilian J Anesthesiol..

[29] Boulton AJ (2008). The diabetic foot: grand overview, epidemiology and pathogenesis. Diabetes Metab Res Rev..

[30] Garbino JA, Marques WJ, Alves ED, Ferreira TL, Ferreira IN (2014). Neuropatia da hanseníase. Hanseníase: avanços e desafios.

[31] Lavery L.A., Peters E.J.G., Armstrong D.G. (2008). What are the most effective interventions in preventing diabetic foot ulcers?. Int Wound J..

[32] Krasner D.L., Rodeheaver G.T., Sibbald R.G. (2007). Chronic wound care: A clinical source book for healthcare professionals.

[33] Brasil (2008). Ministério da Saúde, Secretaria de Vigilância em Saúde, Departamento de Vigilância Epidemiológica. Manual de condutas para tratamento de úlceras em hanseníase e diabetes. 2. ed.. Brasília: Ministério da Saúde.

[34] (2016). Brasil. Ministério da Saúde, Secretaria de Vigilância em Saúde. Diretrizes para vigilância, atenção e eliminação da Hanseníase como problema de saúde pública: manual técnico-operacional.

[35] Frade M.A., Nogueira-Barbosa M.H., Lugão H.B., Furini R.B., Marques Júnior W., Foss N.T. (2013). New sonographic measures of peripheral nerves: a tool for the diagnosis of peripheral nerve involvement in leprosy. Mem Inst Oswaldo Cruz..

[36] Lugão H.B., Nogueira-Barbosa M.H., Marques W, Foss NT, Frade MA (2015). Asymmetric nerve enlargement: a characteristic of leprosy neuropathy demonstrated by ultrasonography. PLoS Negl Trop Dis..

[37] Lima P.O., Cunha F.M., Gonçalves H.S., Aires M.A., De Almeida R.L., Kerr L.R. (2016). Correlation between clinical tests and electroneuromyography for the diagnosis of leprosy neuropathy. Lepr Rev..

[38] Reinar L.M., Forsetlund L., Lehman L.F., Brurberg K.G. (2019). Interventions for ulceration and other skin changes caused by nerve damage in leprosy. Cochrane Database Syst Rev..

[39] (2008). Brasil. Ministério da Saúde, Secretaria de Vigilância em Saúde, Departamento de Vigilância Epidemiológica. Manual de adaptações de palmilhas e calçados.

[40] Sacco I.C.N., Sartor C.D., Gomes A.A., João S.M.A., Cronfli R. (2007). Assessment of motor sensory losses in the foot and ankle due to diabetic neuropathy. Rev Bras Fisioter..

[41] Santos A.A., Bertato F.T., Montebelo M.I.L., Guirro E.C.O. (2008). Effect of proprioceptive training among diabetic women. Rev Bras Fisioter..

[42] Sartor C.D., Watari R., Pássaro A.C., Picon A.P., Hasue R.H., Sacco I.C. (2012). Effects of a combined strengthening, stretching and functional training program versus usual-care on gait biomechanics and foot function for diabetic neuropathy: a randomized controlled trial. BMC Musculoskelet Disord..

[43] Kluding P.M., Pasnoor M., Singh R., Jernigan S., Farmer K., Rucker J. (2012). The effect of exercise on neuropathic symptoms, nerve function, and cutaneous innervation in people with diabetic peripheral neuropathy. J Diabetes Complications..

[44] Kruse R.L., Lemaster J.W., Madsen R.W. (2010). Fall and balance outcomes after an intervention to promote leg strength, balance, and walking in people with diabetic peripheral neuropathy: “feet first” randomized controlled trial. Phys Ther..

[45] Vuerstaek J.D., Reeder S.W., Henquet C.J., Neumann H.A. (2010). Arteriolosclerotic ulcer of Martorell. J Eur Acad Dermatology Venereol..

[46] Hafner J., Nobbe S., Partsch H., Läuchli S., Mayer D., Amann-Vesti B. (2010). Martorell hypertensive ischemic leg ulcer: a model of ischemic subcutaneous arteriolosclerosis. Arch Dermatol..

[47] Alavi A., Mayer D., Hafner J., Sibbald R.G. (2012). Martorell hypertensive ischemic leg ulcer: an underdiagnosed Entity©. Adv Skin Wound Care..

[48] Dagregorio G., Guillet G. (2006). A retrospective review of 20 hypertensive leg ulcers treated with mesh skin grafts. J Eur Acad Dermatol Venereol..

[49] Guisado Muñoz S, Conde Montero E, de la Cueva Dobao P (2019). Punch grafting for the treatment of martorell hypertensive ischemic leg ulcer. Actas Dermosifiliogr..

[50] Shelling M.L., Federman D.G., Kirsner R.S. (2010). Clinical approach to atypical wounds with a new model for understanding hypertensive ulcers. Arch Dermatol..

[51] De Andrés J., Villanueva V.L., Mazzinari G., Fabregat G., Asensio J.M., Monsalve V. (2011). Use of a spinal cord stimulator for treatment of martorell hypertensive ulcer. Reg Anesth Pain Med..

[52] Malhi H.K., Didan A., Ponosh S., Kumarasinghe S.P. (2017). Painful leg ulceration in a poorly controlled hypertensive patient: a case report of martorell ulcer. Case Rep Dermatol..

[53] Hafner J (2016). Calciphylaxis and martorell hypertensive ischemic leg ulcer: same pattern - one pathophysiology. Dermatology..

[54] Kolios A.G., Hafner J., Luder C., Guenova E., Kerl K., Kempf W. (2018). Comparison of pyoderma gangrenosum and Martorell hypertensive ischaemic leg ulcer in a Swiss cohort. Br J Dermatol..

[55] Schultz G.S., Sibbald R.G., Falanga V., Ayello E.A., Dowsett C., Romanelli M. (2003). Wound bed preparation: a systematic approach to wound management. Wound Repair Regen..

[56] Schultz G.S., Barillo D.J., Mozingo D.W., Chin G.A. (2004). Wound bed preparation and a brief history of TIME. Int Wound J..

[57] Moore Z., Dowsett C., Smith G., Atkin L., Bain M., Schutz G.S. (2019). TIME CDST: an updated tool to address the current challenges in wound care. J Wound Care..

[58] Leaper D.J., Schultz G., Carville K., Fletcher J., Swanson T., Drake R. (2012). Extending the TIME concept: what have we learned in the past 10 years? (*). Int Wound J..

[59] Colenci R, Abbade LPF (2018). Fundamental aspects of the local approach to cutaneous ulcers. An Bras Dermatol..

[60] Fernandez R., Griffiths R. (2012). Water for wound cleansing. Cochrane database Syst Rev..

[61] Eberlein T., Assadian O. (2010). Clinical use of polihexanide on acute and chronic wounds for antisepsis and decontamination. Skin Pharmacol Physiol..

[62] Vermeulen H., Westerbos S.J., Ubbink D.T. (2010). Benefit and harm of iodine in wound care: a systematic review. J Hosp Infect..

[63] Falanga V., Brem H., Ennis W.J., Wolcott R., Gould L.J., Ayello E.A. (2008). Maintenance debridement in the treatment of difficult-to-heal chronic wounds. Recommendations of an expert panel. Ostomy Wound Manage..

[64] Strohal R., Dissemond J., Jordan O’Brien J., Piaggesi A., Rimdeika R., Young T. (2013). EWMA Document: Debridement: An updated overview and clarification of the principle role of debridement. J Wound Care..

[65] Wolcott R.D., Rumbaugh K.P., James G., Schultz G., Philips P., Yang Q. (2010). Biofilm maturity studies indicate sharp debridement opens a time-dependent therapeutic window. J Wound Care..

[66] Gethin G., Cowman S., Kolbach D.N. (2015). Debridement for venous leg ulcers. Cochrane database Syst Rev..

[67] Patry J., Blanchette V. (2017). Enzymatic debridement with collagenase in wounds and ulcers: a systematic review and meta-analysis. Int Wound J..

[68] Oliveira H.L., Fleming M.E., Silva P.V., Paula G.R., Futuro D.O., Velarde G.C. (2014). Influence of papain in biofilm formed by methicillin-resistant Staphylococcus epidermidis and methicillin-resistant Staphylococcus haemolyticus isolates. Braz J Pharm Sci..

[69] Onesti M.G., Fioramonti P., Fino P., Sorvillo V., Carella S., Scuderi N. (2016). Effect of enzymatic debridement with two different collagenases versus mechanical debridement on chronic hard-to-heal wounds. Int Wound J..

[70] Figueiredo Azevedo F., Santanna L.P., Bóbbo V.C., Libert E.A., Araújo E.P., Abdalla Saad M. (2017). Evaluating the Effect of 3% Papain Gel Application in Cutaneous Wound Healing in Mice. Wounds..

[71] Doerler M., Reich-Schupke S., Altmeyer P., Stücker M. (2012). Impact on wound healing and efficacy of various leg ulcer debridement techniques. J Dtsch Dermatol Ges..

[72] Hurlow J., Blanz E., Gaddy J.A. (2016). Clinical investigation of biofilm in non-healing wounds by high resolution microscopy techniques. J Wound Care..

[73] Malone M., Bjarnsholt T., McBain A.J., James G.A., Stoodley P., Leaper D. (2017). The prevalence of biofilms in chronic wounds: a systematic review and meta-analysis of published data. J Wound Care..

[74] Sibbald R.G., Orsted H., Schultz G.S., Coutts P., Keast D., International Wound Bed Preparation Advisory Board (2003). Preparing the wound bed 2003: focus on infection and inflammation. Ostomy Wound Manage..

[75] Bonham PA (2009). Swab Cultures for Diagnosing Wound Infections: a literature review and clinical guideline. J Wound Ostomy Continence Nurs..

[76] Snyder R.J., Bohn G., Hanft J., Harkless L., Kim P., Lavery L. (2017). Wound biofilm: current perspectives and strategies on biofilm disruption and treatments. Wounds..

[77] Wolcott R.D., Cox S. (2013). More effective cell-based therapy through biofilm suppression. J Wound Care..

[78] Raju R., Kethavath S.N., Sangavarapu S.M., Kanjarla P. (2019). Efficacy of cadexomer iodine in the treatment of chronic ulcers: a randomized, multicenter, controlled trial. Wounds..

[79] Jaffe L., Wu SC (2019). Dressings, topical therapy, and negative pressure wound therapy. Clin Podiatr Med Surg..

[80] Walker R.M., Gillespie B.M., Thalib L., Higgins N.S., Whitty J.A. (2017). Foam dressings for treating pressure ulcers. Cochrane database Syst Rev..

[81] Kaya A.Z., Turani N., Akyüz M. (2005). The effectiveness of a hydrogel dressing compared with standard management of pressure ulcers. J Wound Care..

[82] Percival SL, McCarty SM (2015). Silver and alginates: role in wound healing and biofilm control. Adv Wound Care (New Rochelle).

[83] Sood A, Granick MS, Tomaselli NL (2014). Wound dressings and comparative effectiveness data. Adv Wound Care (New Rochelle)..

[84] Tate S., Price A., Harding K. (2018). Dressings for venous leg ulcers. BMJ..

[85] Gao A.L., Cole J.G., Stoecker W.V. (2017). Unna boot central gauze technique for chronic venous leg ulcers. Dermatol Online J..

[86] Sabo M., Le L., Yaakov R.A., Carter M., Serena T.E. (2018). A post-marketing surveillance study of chronic wounds treated with a native collagen calcium alginate dressing. Ostomy Wound Manage..

[87] Aziz Z., Cullum N. (2015). Electromagnetic therapy for treating venous leg ulcers. Cochrane database Syst Rev..

[88] Flemming K., Cullum N. (2000). Laser therapy for venous leg ulcers. Cochrane Database Syst Rev..

[89] Dumville J.C., Land L., Evans D., Peinemann F. (2015). Negative pressure wound therapy for treating leg ulcers. Cochrane Database Syst Rev..

[90] Cullum N., Liu Z. (2017). Therapeutic ultrasound for venous leg ulcers. Cochrane Database Syst Rev..

